# Changing landscapes drive dietary diversification in Asian elephants

**DOI:** 10.1038/s41598-026-41675-0

**Published:** 2026-03-12

**Authors:** Nurfatin Batrisyia, Noor Fatihah Najihah Arazmi, Muhammad Iqbal Md Jamaluddin, Ummi Nur Syafiqah Daud, Nor Adibah Ismail, Aisah Md Shukor, Muhammad Fadlli Ab Yazi, Shukor Md Nor, Mohammad Saiful Mansor

**Affiliations:** 1 Department of Biological Sciences and Biotechnology, Faculty of Science and Technology, Universiti Kebangsaan Malaysia, 43600 UKM Bangi, Selangor Malaysia; 2 Department of Earth Sciences and Environment, Faculty of Science and Technology, Universiti Kebangsaan Malaysia, 43600 UKM Bangi, Selangor Malaysia; 3 TNB Research, No. 1, Kawasan Institusi Penyelidikan, Jalan Ayer Itam, 43000 Kajang, Selangor Malaysia; 4 Department of Wildlife and National Parks (PERHILITAN) Peninsular Malaysia, KM 10, Jalan Cheras, 56100 Kuala Lumpur, Malaysia; 5 Pelan Urus Services, Suite 3, Bangi Gateway, Seksyen 15, 43650 Bandar Baru Bangi, Selangor Malaysia

**Keywords:** Dietary diversity, DNA metabarcoding, *Elephas maximus*, Land-use change, Next-generation sequencing, Biological techniques, Biotechnology, Ecology, Molecular biology, Plant sciences, Zoology, Ecology, Environmental sciences

## Abstract

Rapid changes in land-use patterns bring significant challenges to wildlife, particularly for large herbivores, such as Asian elephants (*Elephas maximus*). Despite the impact of land-use change being widely studied globally, its effect on the dietary patterns of umbrella species, such as the Asian elephant, remains limited. Therefore, this study employed high-throughput *trnL* DNA metabarcoding to characterise and compare the diets of Asian elephants across two distinct landscapes: (1) northeast Peninsular Malaysia, a landscape undergoing large-scale development and logging (development-logging landscapes, DLL), and (2) southern Peninsular Malaysia, an oil palm-dominated landscape with remnant forests without major logging or land-use changes (oil palm-forest landscapes, OPFL). We analysed 60 individual faecal samples, yielding 1,737,956 high-quality sequences for the DLL and 1,454,807 for the OPFL. Analysis of frequency of occurrence (FOO) and relative read abundance (RRA) revealed a significant variation in elephant diets between the two landscapes, with the DLL exhibiting higher richness and diversity than the OPFL. This study demonstrates the dietary flexibility of Asian elephants, showing their ability to adapt to environmental changes in DLL by modifying their feeding habits according to available food resources. The findings also reveal the diet-related dimensions of human–elephant conflict across contrasting landscapes and highlight the need for strategic landscape management, including habitat restoration and ecological corridors, to reduce conflict and support long-term conservation.

## Introduction

The native population of the Asian elephant (*Elephas maximus*) in Peninsular Malaysia is estimated to be between 1223 and 1677 individuals, distributed across six states: Kelantan, Pahang, Terengganu, Kedah, Perak, and Johor^1, 2^. Since 1986, the Asian Elephant has been classified as endangered (EN) in the International Union for Conservation of Nature (IUCN) Red List of Threatened Species^3^. Legislation enacted by the Malaysian government, the Wildlife Conservation Act 2010 (Act 716), provides legal protection for the Asian elephant in Peninsular Malaysia, classifying the species as totally protected in the Malaysian Red List of endangered species^4^. The Managed Elephant Ranges (MERs), centred within the Central Forest Spine (CFS), a network of interconnected forested landscapes spanning Peninsular Malaysia, encompass three major forest complexes: Belum-Temenggor, Taman Negara, and Endau-Rompin. Elephantpopulations predominantly range within these forest complexes and surrounding forests designated under the MERs framework ^1, 2, 5, 6^, but also occur beyond these areas.

Asian elephants are mixed-feeding megaherbivores whose natural diet reflects the structural and compositional diversity of vegetation, comprising graminoids, browse and fruiting plants. Across their range, studies consistently report frequent consumption of taxa such as *Ficus* spp., bamboo species (*Dendrocalamus* spp.), wild bananas (*Musa* spp.), and grasses, indicating a broad dietary breadth across heterogeneous tropical plant communities^7, 8, 9^. However, land-use change alters vegetation structure and movement patterns, leading to variation in habitat use and ranging behaviour, and can modify dietary composition through adaptive strategies. For megafauna such as elephants, these pressures often manifest as habitat loss and fragmentation, constraining natural ranging and increasing reliance on human-modified landscapes. As the largest terrestrial mammals, elephants require vast resources to sustain their health and ecological functions^10, 11^, yet food availability and dietary composition remain closely linked to habitat integrity, which in turn shapes their foraging behaviour^9^. Habitat fragmentation and anthropogenic disturbances disrupt their natural foraging patterns, potentially leading to nutritional stress, population declines and cascading effects on ecosystems^12^. In this context, optimal foraging theory predicts that large herbivores maximise energy intake while minimising effort by selecting foods based on availability and nutritional value^13^. Consequently, elephants preferentially consume high-nutrient plant species when resources are abundant but broaden their diet to include lower-quality forage when resources become limited^14, 15^.

In Malaysia, the conversion of forests into oil palm plantations and ongoing logging activities has significantly altered the natural habitats of Asian elephants. These landscape changes not only reduce the availability of native vegetation but also force elephants to modify their foraging behaviour. Such responses are further intensified by habitat fragmentation, including the development of linear infrastructure such as roads and railways, which disrupt elephant movement, thereby limiting access to available resources and connected habitats^16^. As a result, elephants often consume less nutritious plant species as they adjust their dietary habits to cope with reduced availability of key food resources^17^. The loss of habitat due to agricultural expansion forces elephants to seek alternative food sources. Elephants living near plantations actively exploit cultivated crops, such as oil palm, banana and other agricultural produce, due to their greater availability and accessibility^18^. Such use of cultivated plants is strongly associated with increased human-elephant conflict (HEC). Despite growing research on the diet composition of Asian elephants, most studies have focused on descriptive analyses^5, 7, 19, 20^, with limited emphasis on how elephants adapt their foraging strategies in response to landscape changes. This remains a critical gap in the understanding of the long-term impacts of dietary variation for elephant health and ecosystem dynamics. Addressing this knowledge gap is crucial for developing effective conservation strategies to ensure the species persistence, particularly in landscapes undergoing rapid anthropogenic change.

In this study, we used DNA metabarcoding to assess the dietary composition of Asian elephants across two landscape types: development-logging landscapes (DLL) and oil palm-forest landscapes (OPFL). We hypothesised that rapid landscape change influences the dietary diversity of Asian elephants, with elephants in DLL exhibiting higher dietary diversity than those in OPFL due to foraging on a wider range of plant species to compensate for reduced availability of preferred forage. Recent logging activities in DLL may lead to reduced diet selectivity, forcing elephants to consume any available plant species, including less-preferred and lower-quality plants, to meet their nutritional requirements. In contrast, elephants in the relatively stable OPFL, where plantations dominate but no major logging is ongoing, may exhibit a more selective diet, relying on the consistent availability of preferred plant species to which they have adapted. Understanding wildlife dietary selection and survival strategies through detailed dietary analysis is crucial for effective wildlife conservation and management.

## Methods

### Study area

**Fig. 1:**
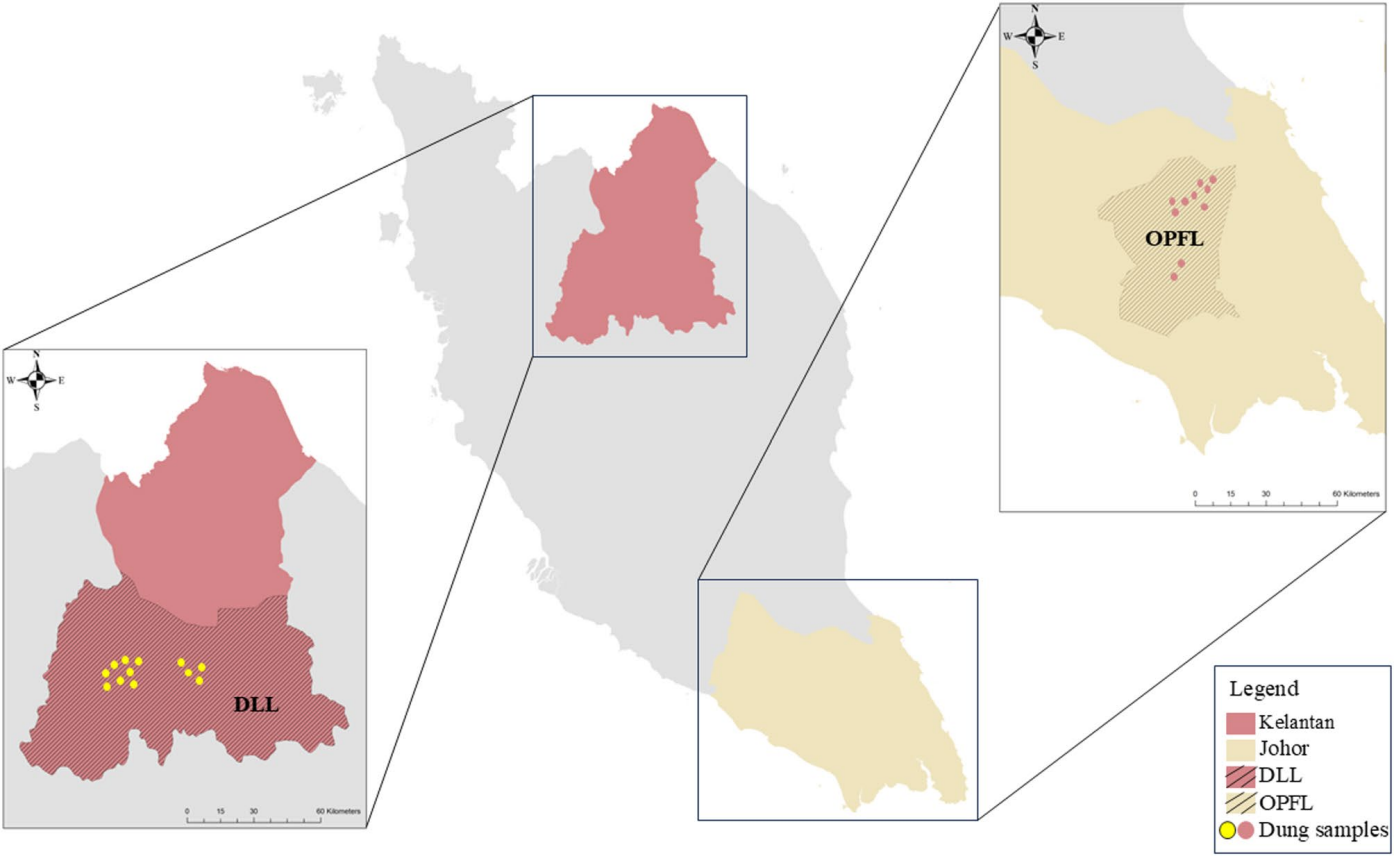
Map of faecal sampling sites for Asian elephants in the development–logging landscape (DLL) in northeast Peninsular Malaysia and the oil palm–forest landscape (OPFL) in southern Peninsular Malaysia. The map was produced using ArcGIS v10.8.2 (ESRI; https://www.esri.com).

Fieldwork was conducted in two landscape types in Peninsular Malaysia: DLL and OPFL (Fig. 1). The DLL are situated in Gua Musang, Kelantan, and represents a heavily modified landscape, characterised by large-scale hydroelectric dam development. Current logging activities involve the dam development zone and the surrounding project area. The forest structure within DLL is heterogenous, consisting of a mosaic of primary and secondary forests with some patches maintaining connectivity to nearby limestone forests. The OPFL of Johor in southern Peninsular Malaysia represent a relatively stable environment. Large-scale deforestation between the 1980s and the early 2000s resulted in extensive conversion of natural forests, leaving most of the landscape now dominated by oil palm plantations, with a narrow forest strip remaining in the central part of Johor that is often used by elephants as a refuge.

### Sample collection

A total of 86 fresh faecal samples were collected across two landscape types in DLL and OPFL from 15 May 2023 until 1 October 2023. Freshly deposited dung piles, characterised by their moist texture, intact structural integrity and distinct odour, were collected to ensure high DNA yield. To prevent contamination, sterile gloves were worn throughout sample collection. Using sterile forceps, the inner layers of whole dung boluses were carefully taken, maximising representativeness while minimising contamination risk. Samples were immediately transferred into sterile 30 mL falcon tubes containing undenatured alcohol (99.9%) and stored at − 20 ℃ in the laboratory until DNA extraction.

### DNA extraction and PCR amplification

DNA was extracted from 86 faecal samples (46 from DLL and 40 from OPFL) for dietary analysis. Genomic DNA was extracted as soon as the samples arrived in the laboratory (5 to 7 days after collection) using QIAamp Fast DNA Stool Mini Kit (Qiagen, Hilden, Germany), following the manufacturer’s protocols with modifications. Faecal samples were crushed in the presence of liquid nitrogen using a mortar and pestle prior to adding InhibiteX buffer to the sample. The incubation period with Buffer AL and proteinase K was increased from 10 to 30 min. Purity of the extracted DNA was evaluated spectrophotometrically, with a Nanodrop™ Lite (Thermo Fisher Scientific, Waltham, MA, USA) using the A_260/280_ OD ratio. The concentration threshold was set at 20 ng with a minimum volume of 20 µL, resulting in 60 samples (30 from DLL and 30 from OPFL) selected. Purified DNA extracts were preserved at − 22 °C prior to library preparation.

The *trnL* intron was amplified using primers targeting the P6 loop, generating an amplicon of approximately 90–100 bp in the first PCR cycle and 100–200 bp in the second nested PCR cycle. The amplification was performed using the *trnL-g* forward (5′-GGGCAATCCTGAGCCAA-3′) and *trnL-h* reverse (5′-CCATTGAGTCTCTGCACCTATC-3′) primers. Both primers were attached to Illumina overhang adapter sequences: forward overhang (5′-TCGTCGGCAGCGTCAGATGTGTATAAGAGACAG-3′) and reverse overhang (5′-GTCTCGTGGGCTCGGAGATGTGTATAAGAGACAG-3′). PCR amplification was conducted in two cycles. The first PCR was carried out in a 50 µl reaction mixture containing 10 µl of 5× PCR buffer, 1 µl of 10 mM dNTP mix, 2.5 µl of each primer (10 µM), 0.5 µl of thermostable DNA polymerase (2 U/µl), and up to 5 µl of genomic DNA (50 ng template), with the final volume adjusted using nuclease-free water. The second PCR (nested PCR) was performed using 1 µl of the first PCR product as a template in a reaction containing 10 µl of 5× PCR buffer, 1 µl of 10 mM dNTP mix, 2.5 µl of each adapter-linked primer (10 µM), 0.5 µl of thermostable DNA polymerase (2 U/µl) and nuclease-free water. PCR cycling conditions for both reactions consisted of an initial denaturation at 95 °C for 3 min, followed by 30 cycles of denaturation at 98 °C for 30 s, annealing at 64 °C for 30 s, extension at 72 °C for 30 s, and a final extension at 72 °C for 5 min. A negative control, consisting of nuclease-free water instead of template DNA, was included during the PCR stage to check for contamination. The amplified PCR products were then verified on a 1.7% TAE agarose gel, run at 100 V for 65 min.

### Bioinformatic processing

Purified amplicons were pooled in equimolar amounts and paired-end sequenced (2 × 150) on an Illumina MiSeq platform (Illumina, San Diego, CA, USA) according to standard protocols. The analysis was conducted following the Quantitative Insights into Microbial Ecology 2 (QIIME2) pipeline 2024.5 using QIIME2 customised program scripts (https://www.docs/qiime2.org/2024.5/)^21^. Raw sequences were subjected to filtering, trimming, denoising and merging using the DADA2 method. Each unique sequence generated was clustered and is referred to as an amplicon sequence variant (ASV) at a 100% similarity threshold. The ASV table generated in QIIME2 was exported and analysed further using Geneious Prime (Biomatters Ltd., New Zealand). The obtained ASV sequences were subjected to BLASTn analysis against the NCBI nucleotide (nt) database (https://www.ncbi.nlm.nih.gov/nucleotide/) using the Megablast algorithm (set to five hits and E-value = 1e-10) to ensure high-confidence matches^22, 23^. BLAST hits with a sequence identity of 100% were retained for subsequent analysis.

For plant taxonomy identification, sequences with ≥ 98% identity that matched a species native to Malaysia were assigned to the species level, whereas non-native species were marked as Not Available (NA). If a query matched ≥ 98% identity but could not be reliably distinguished (i.e., forming a species complex), the sequence was assigned to the genus level rather than to a single species. Queries with sequence identity ranging from 95% to 98% were assigned to the genus level, whereas those with 90–94% identity were classified at the family level^24, 25^. To enhance accuracy, local species distribution data from the Malaysia Biodiversity Information System (MyBIS) and the List of Plant Species in Peninsular Malaysia were used for manual verification.

Relative read abundance (%RRA) and frequency of occurrence (%FOO) were used to measure the plant composition and occurrence. %RRA is the number of sequences of a species category as a percentage of the total species category sequences for a sample, reflecting the relative biomass, while %FOO is the number of samples of a food sequence as a percentage of the total samples. The specific formulae for RRA and FOO are as follows:

$$\:\%\:{RRA}_{i}={\sum\:}_{k=1}^{N}\frac{{n}_{i,k}}{{\sum\:}_{i=1}^{T}{n}_{i,k}}\times\:100\mathrm{\%}$$

where *N* is the total number of faecal samples; *T* is the total number of species categories, and $\:{n}_{i,k}$ is the number of sequences of plant species *i* in sample *k.*

$$\:\%\:FOO=\:\frac{{N}_{i}}{N}\:\times\:100\%$$

where *Ni* is the number of faecal samples containing plant species *I* and *N* is the total number of faecal samples.

### Statistical analysis

The Shannon index, Simpson’s diversity index, Evenness index, Dominance index, Margalef index, and Berger–Parker index was calculated using the *diversity* function in PAST software (version 4.03) (Hammer and Harper 2001) to quantify dietary diversity, dominance and evenness in elephant diets. Results were visualised using R (version 4.4.1) (R Development Core Team 2024). A chord diagram was generated using the *circlize* package^26^ to illustrate dietary composition between elephants in different landscapes. Differences in the dietary compositions of Asian elephants in DLL and OPFL were evaluated based on the Bray–Curtis distance, and the beta diversity results was visualised using non-metric multidimensional scaling (NMDS). The statistical significance of differences in the dietary composition was tested using permutational multivariate analysis of variance (PERMANOVA) with 999 permutations. The analysis was conducted in R (version 4.4.1) using the *adonis2()* function from the *vegan* package. A Bray–Curtis dissimilarity matrix of dietary composition was constructed based on these taxa and tested for differences in dietary composition between the two groups (DLL vs. OPFL^27^.

## Results

High-throughput DNA metabarcoding of the *trnL* region generated a total of 1,857,681 and 1,581,620 raw sequences from all faecal samples collected in DLL and OPFL, respectively. After quality filtering, chimera removal, and dereplication, 1,737,956 high-quality reads were retained for DLL and 1,454,807 for OPFL. Taxonomic assignment of the processed sequences identified 163 ASVs for DLL and 154 ASVs for OPFL. The dietary composition of Asian elephants in DLL exhibited greater taxonomic richness, with 103 plant species identified across 114 genera from 50 families whereas in OPFL, 69 plant species were identified across 73 genera belonging to 38 families (Table S1).

**Fig. 2:**
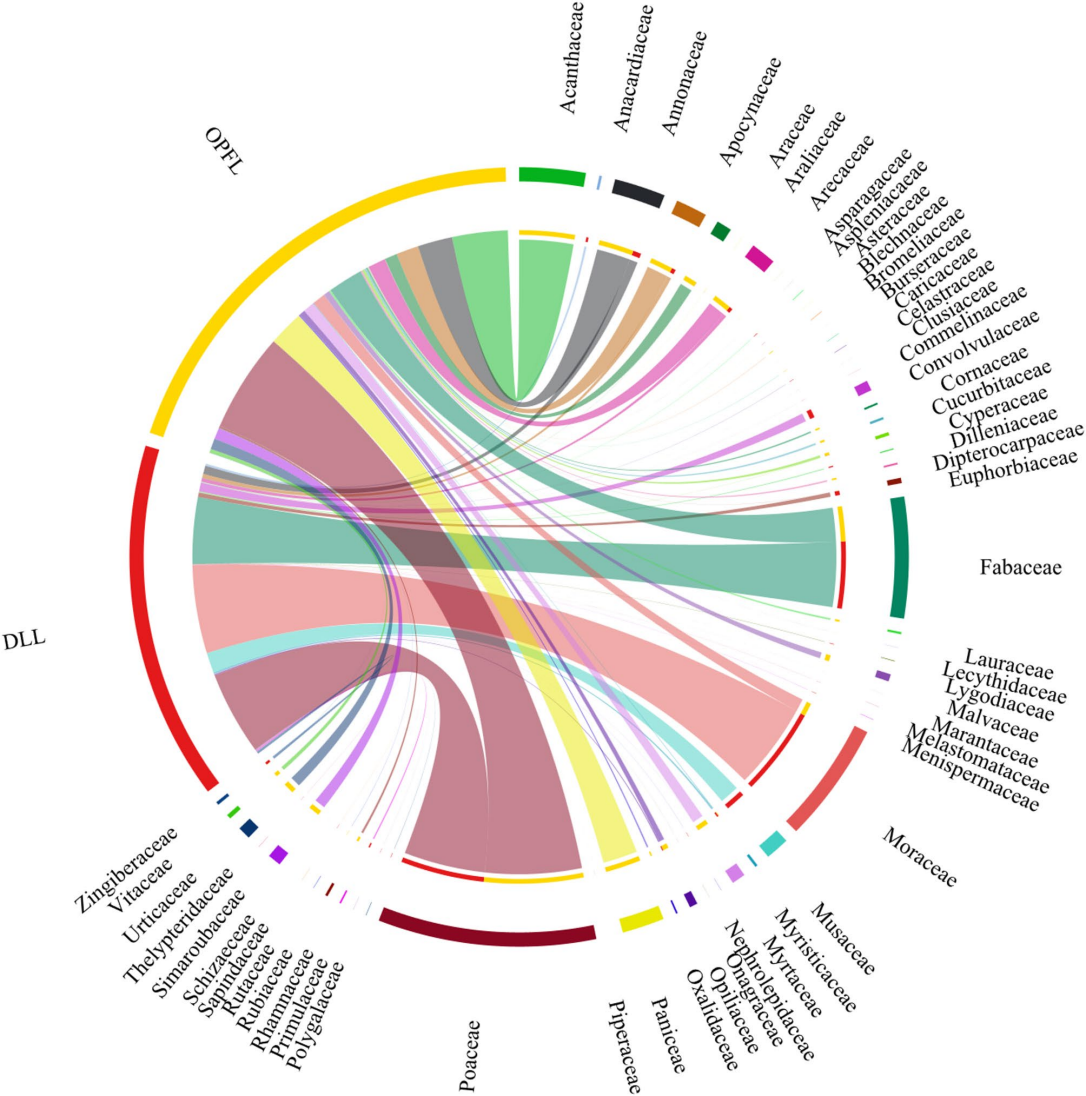
Relative read abundance (%RRA) of plant families detected in faecal samples of Asian elephants from two landscapes: Development-logging landscape (DLL; Red) and Oil palm-forest landscape (OPFL; Yellow). Each colored segment represents a plant family, with chords linking families to the landscapes.

**Fig. 3:**
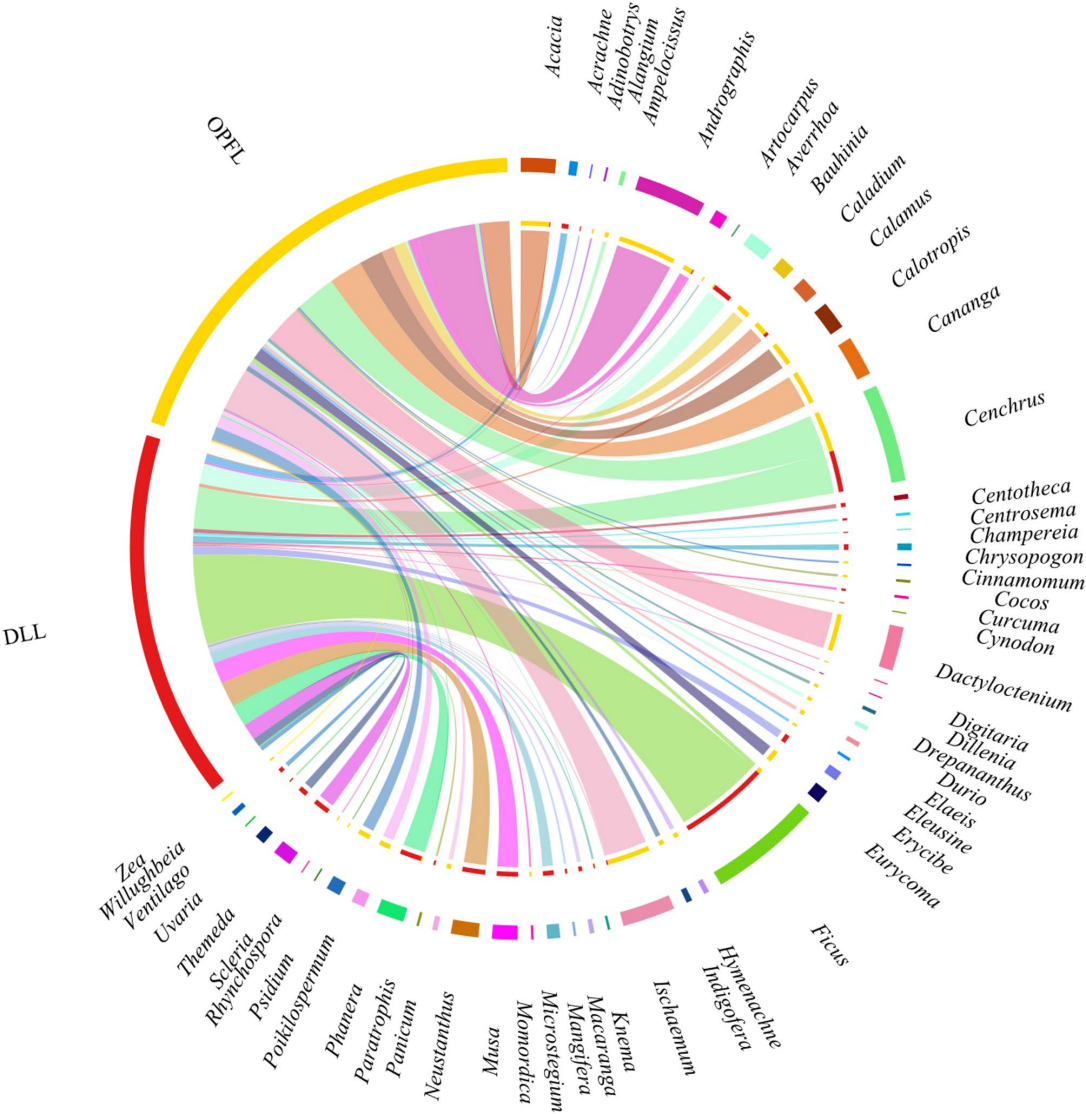
Chord diagram showing relative read abundance (%RRA) of plant genus detected in faecal samples of Asian elephants from two landscapes: Development-logging landscape (DLL; Red) and Oil palm-forest landscape (OPFL; Yellow). Each colored segment represents a plant genus, with chords linking genera to the landscapes.

**Fig. 4:**
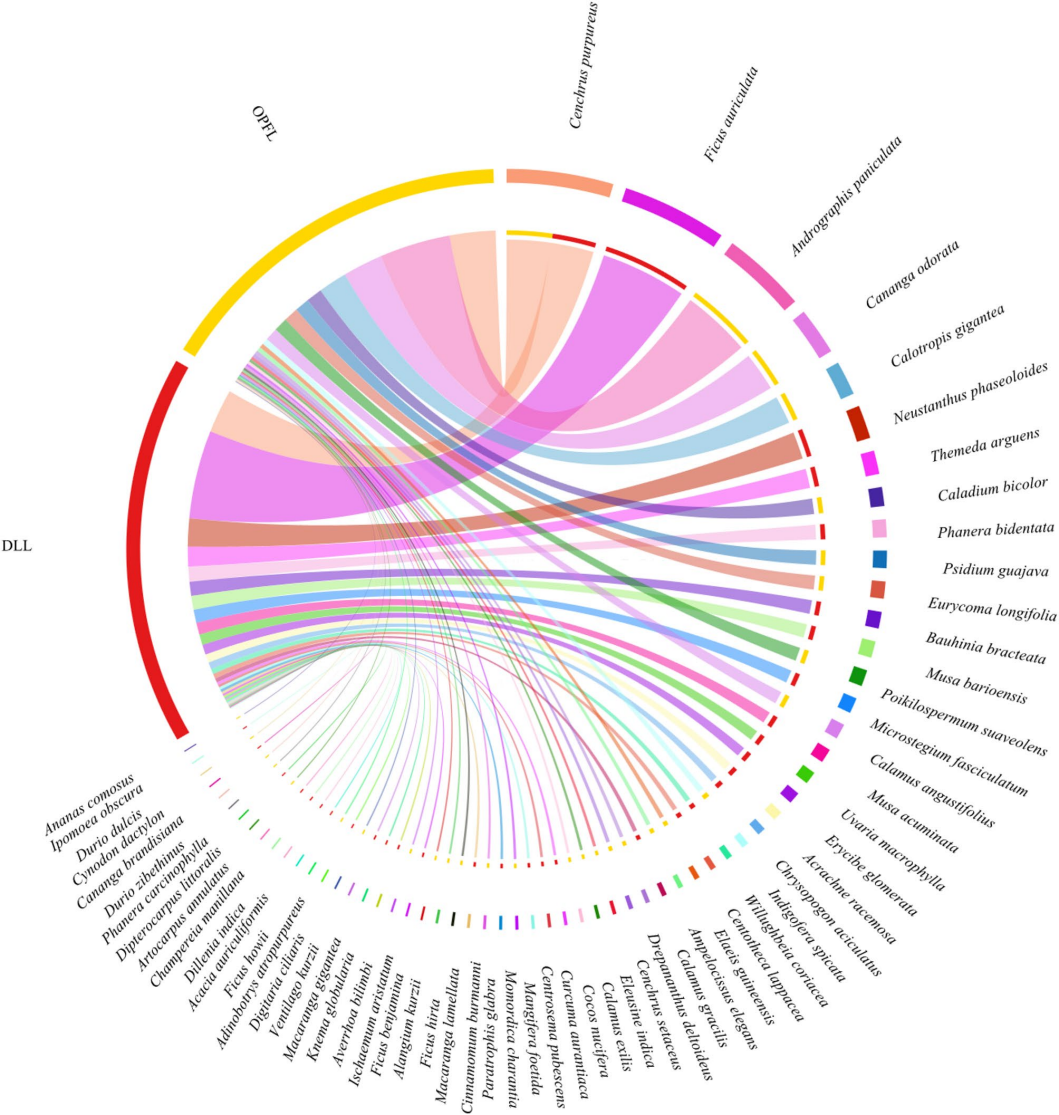
Chord diagram showing relative read abundance (%RRA) of plant species detected in faecal samples of Asian elephants from two landscapes: Development-logging landscape (DLL; Red) and Oil palm-forest landscape (OPFL; Yellow). Each colored segment represents a plant species, with chords linking plant species to each landscape.

Of the 63 plant families identified, Poaceae (%RRA = 26.47%), Fabaceae (%RRA = 14.66%) and Moraceae (%RRA = 14.64%) were the most dominant in the diet of Asian elephants across both landscapes (Fig. 2). However, their relative abundance varied between landscapes, with DLL exhibiting a higher dominance of Moraceae (%RRA = 29.26%), followed by Poaceae (%RRA = 27.80%) and Fabaceae (%RRA = 22.11%). In contrast, OPFL displayed a more balanced distribution, with Poaceae (%RRA = 25.44%), Acanthaceae (%RRA = 14.22%) and Panicaceae (%RRA = 9.1%) contributing significantly to the diet (Figure. 2). At the genus level, dietary composition was significantly different between DLL and OPFL (Fig. 3). In DLL, *Ficus* (Moraceae) dominated the diet (%RRA = 28.1%), followed by *Cenchrus* (%RRA = 13.0%) and *Neustanthus* (%RRA = 7.23%). Other notable genera included *Phanera* (%RRA = 6.84%) and *Musa* (%RRA = 6.67%). In OPFL, *Andrographis* (%RRA = 14.73%), *Ischaemum* (%RRA = 10.83%) and *Cenchrus* (%RRA = 10.26%) were dominant, while *Ficus* was significantly lower than in DLL (%RRA = 1.18%). At the species level, *Ficus auriculata* (Moraceae) was the most dominant species in DLL (%RRA = 26.19%), followed by *Cenchrus purpureus* (Poaceae, %RRA = 13.00%) and *Neustanthus phaseoloides* (Fabaceae, %RRA = 8.30%) (Fig. 4). In OPFL, the dietary composition was characterised by higher contributions of *Andrographis paniculata* (Acanthaceae, %RRA = 22.50%) and *Cenchrus purpureus* (Poaceae, %RRA = 14.46%) as key contributors, while *Cananga odorata* (Annonaceae, %RRA = 12.63%) remained prevalent in the diet of elephants in OPFL.

Despite these differences, 130 plant species were shared across landscapes, forming the core diet of Asian elephants. However, DLL supported a greater number of unique species (90), including *Ficus auriculata* and *Musa acuminata*, which were absent in OPFL. Conversely, OPFL contained 54 unique species, such as *Caladium bicolor* and *Elaeis guineensis*. The greater number of unique taxa detected in DLL, relative to OPFL, provides an indirect indication of higher available plant diversity within this landscape. The %FOO of dietary plant taxa also showed a significant variation between landscapes, with elephants in DLL consuming a greater diversity of taxa compared to those in OPFL. In DLL, a total of 16 plant taxa were identified with an FOO ≥ 0.8, including dominant species such as *Ficus auriculata* and *Cenchrus purpureus* (FOO = 1.0), followed by *Calamus gracilis* (FOO = 0.8). The dietary profile in DLL suggests a reliance on early-successional and pioneer plant species commonly found in fragmented forest edges and regenerating landscapes. Conversely, in OPFL, a total of 7 plant taxa were identified with a FOO ≥ 0.8, reflecting a more selective dietary pattern dominated by cultivated and plantation-associated species, such as *Durio zibethinus*, *Cananga odorata and Zea mays* (FOO = 1.0). The prevalence of these species in OPFL highlights the elephants’ adaptation to human-modified environments, with a focus on high-energy food sources such as *Durio* and *Zea*, and readily available food sources.

**Fig. 5:**
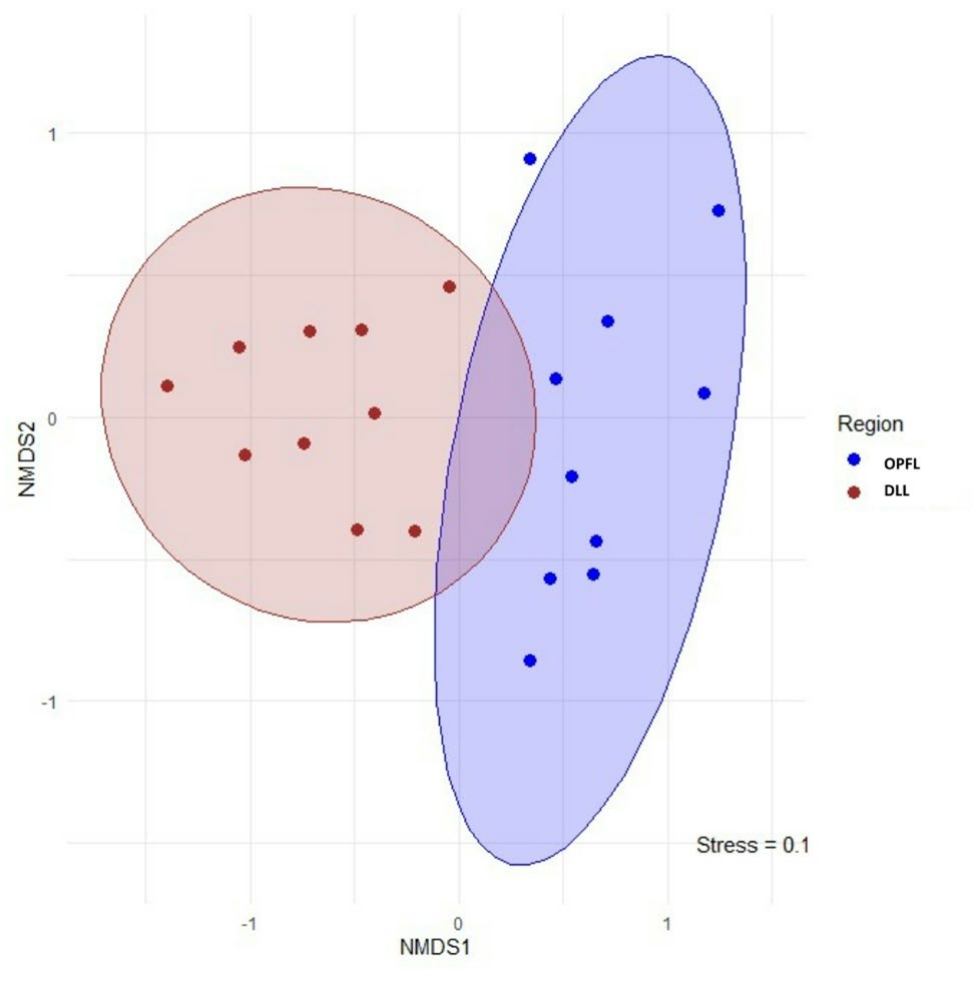
Comparison of dietary variations using non-metric multidimensional scaling (NMDS) in Asian elephants in different regions.

**Fig. 6:**
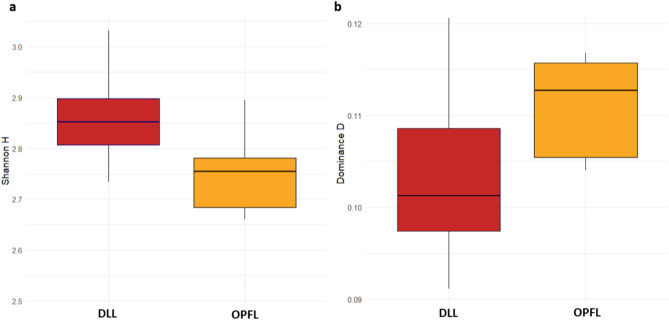
Alpha diversity indices of dietary composition in Asian elephants across two landscapes (DLL; red) and (OPFL; yellow) (**a**) Shannon index); (**b**) dominance index.

Significant differences in elephant dietary composition were observed between the two landscapes (PERMANOVA: F = 6.235, p < 0.05), with DLL exhibiting higher dietary diversity than OPFL. Comparison of dietary variations using non-metric multidimensional scaling (NMDS) based on the Bray–Curtis distance confirmed the spatial variation in diet, illustrating significant dissimilarities in plant consumption (stress = 0.112; Fig. 5). Alpha diversity analysis using the Shannon index (H’), a measure of species diversity that accounts for both richness and evenness, indicated intergroup differences in plant availability and habitat composition, with a higher index in DLL (H′ = 2.861) than in OPFL (H′ = 2.711) (Fig. 6a) and higher dominance index of OPFL (Fig. 6b). Further analysis of plant species richness using the Margalef index and Chao-1 estimator supports this pattern, with DLL exhibiting higher species richness (7.407) than OPFL (4.962).

## Discussion

High-throughput DNA metabarcoding revealed significant differences in elephant dietary composition between the two landscapes, with elephants in development-logging landscapes (DLL) exhibiting higher dietary diversity than those in oil palm-dominated landscapes (OPFL). This highlights the importance of landscape configuration in shaping dietary composition, suggesting that elephants inhabiting structurally distinct environments utilise different plant assemblages. Results from the DLL reflect broader ecological responses in which habitat fragmentation and land-use changes force large herbivores to expand their diets to meet nutritional requirements^5, 28, 29^. This dietary flexibility observed in this study indicates the capacity of Asian elephants to utilise a wider range of available plant resources across heterogeneous landscapes. Similar patterns have been reported in Bornean populations, where elephants exhibit selective foraging as an adaptive response to altered landscapes, prioritising abundant, high-biomass plant resources over plant quality^30^. However, increased use of agricultural areas in degraded landscapes has been linked to elevated human-elephant conflict^31^. These patterns correspond with movement ecology studies showing that elephants inhabiting disturbed forests traverse a wider range of habitat types, consequently increasing exposure to a broader diversity of plant resources^32^. In contrast, elephants in the OPFL consumed a narrower range of plant taxa, indicating a more predictable and less variable diet over time and reflecting long-term adaptation to a landscape with limited resource availability, where cultivated and forest-edge plant species constitute the main accessible forage.

Elephants in DLL, characterised by ongoing logging and infrastructure development for dam projects, as well as surrounding logging activities for forest plantation projects, occur within landscapes where forest structure and vegetation composition have been substantially modified. Recent land-use changes in DLL forced elephants to become opportunistic feeders, with the mosaic of habitat types providing a wider variety of plant species for foraging. The presence of mixed landscapes, including grasslands, secondary forests, and regenerating vegetation, allows elephants in DLL to access a broader range of food resources. Resource heterogeneity in fragmented systems is widely recognised as a key factor influencing large herbivore foraging patterns^33^. This aligns with the optimal foraging theory, which suggests that animals adjust their diet and movement based on resource availability to optimise food intake^13^. Similarly, opportunistic diet selection of elephants in DLL mirrors the selective foraging behaviour documented in English et al.^30^, where Bornean elephants prioritised readily available plant resources in modified forest and edge habitats rather than plant quality traits. As a result, elephants may utilise a broader range of plant species due to limited food sources or expand their movement beyond their original roaming areas, subsequently enlarging their home range. This aligns with a study by^28, 34^, which reported that elephants in conflict-prone areas may travel longer distances in search of food. This indicates that protected areas alone are inadequate for wide-ranging megaherbivores, as elephant home ranges frequently extend into disturbed habitat which supports the interpretation that DLL elephants depend on a complex mosaic of habitats beyond formal reserve boundaries.

Over the past few decades, large tracts of lowland rainforest that are the main foraging grounds for elephants have been converted into oil palm plantations, rubber estates and urban areas^35^. Although reported as a high HEC zone^36^, elephants in OPFL, a landscape dominated by oil palm plantations with a strip of forest in the central and northern regions, exhibited a more stable and selective diet. The dominance of plants from family Arecaceae (*Elaeis guineensis*) and Poaceae (*Cenchrus* sp.) in the diet of Asian elephants in OPFL with FOO = 1.0 indicates that elephants in disturbed habitats rely on both forest-edge species and early-successional plants, which are often abundant in fragmented habitats. This aligns with the study by Chen et al.^37^ who observed that elephants in China mainly consumed pioneer species and agricultural crops because of the loss of native vegetation. Both *Durio* and *Polyalthia* in the elephant’s diet are commonly associated with plantation landscapes in Peninsular Malaysia. This suggests a greater reliance on plantation-associated species in OPFL compared to DLL, consistent with the documented land-use characteristics of these landscapes. The more homogeneous landscape in OPFL has led to narrower and more selective elephant diets, potentially due to long-term adaptation to the stable availability of limited plant resources as reflected by the lower number of unique plant taxa detected in OPFL compared to DLL, suggesting reduced vegetation heterogeneity and overall forage availability within plantation-dominated systems^5, 37, 38^. In the oil palm-dominated landscapes, the availability of energy-rich and easily accessible plant resources may encourage elephants to forage within human-modified areas for foraging^6, 12, 39^. In this study, the diet composition in OPFL included cultivated crops, indicating that elephants actively feed on these plants within such landscapes. Repeated exposure to cultivated crops likely reinforces elephants’ preference for these species, leading to habitual crop-raiding behaviour. The abundance of cultivated species in OPFL diet suggests a substantial reliance on human-modified habitats for forage.This dietary pattern suggests that elephants in oil palm-dominated landscapes selectively forage on crops and fruit-bearing trees, leading to a more specialised diet^40^. Similarly, elephants in agricultural areas frequently consume oil palm fruits and other plantation-associated plants, which are readily available and rich in energy^6^. These species are not only energy-rich but also widely distributed in agricultural and plantation areas, making them easily accessible to elephants. These patterns align with findings that elephants in stable environments exhibit more selective foraging behaviour, focusing on preferred species that offer better nutritional value^5, 41^.

### Implications for management

Human–elephant conflict (HEC) remains a major conservation and socio-economic challenge in Malaysia, particularly in landscapes where elephant range areas overlap with agricultural expansion. Habitat loss and infrastructure development disrupt elephant movement and reduce natural forage availability, increasing the likelihood of elephants entering agricultural fields and settlements in search of essential resources ^39, 42^. Between 2020 and 2024, nearly 5000 HEC cases were reported nationwide, causing losses of about US$10.1 million, with Kelantan and Johor being the most affected states and Kelantan recorded the highest number of elephant translocations involving 98 individuals since 2020^43, 44,45^. In fragmented systems where natural forage is unevenly distributed or locally reduced, cultivated fields may offer higher energetic returns despite elevated human-associated risks. Empirical studies consistently identify forest edges and human-dominated interfaces as significant predictors of HEC intensity, highlighting the role of landscape configuration in structuring conflict risk^46,47^. Repeated exposure to predictable crop resources may reinforce crop-use behaviour over time, contributing to persistent conflict dynamics. Our results indicate that dietary composition differs between development–logging landscapes (DLL) and oil palm–forest landscapes (OPFL), demonstrating that landscape configuration influences resource selection. In DLL, where structural disturbance alters vegetation composition, elephants exhibited broader dietary diversity, consistent with compensatory foraging in response to reduced availability of preferred plants, potentially accompanied by expanded and modified ranging movements that elevate conflict risk. In contrast, in OPFL landscapes, where anthropogenic food sources are spatially predictable, repeated crop use may increase reliance on high-conflict areas, particularly during oil palm replantation cycles when felled palms are chipped, creating concentrated and easily accessible forage. Dietary plasticity may buffer elephants against habitat disturbance; however, under certain landscape configurations, it may also increase exposure to anthropogenic risk. Restoring habitat connectivity through ecological corridors is widely recognised as a strategy for reducing fragmentation effects by re-establishing movement pathways between forest blocks^48, 49^. However, structural connectivity alone is insufficient. Corridor effectiveness depends on habitat quality and the alignment between forage availability and elephants’ feeding ecology. Asian elephants rely predominantly on grasses and other monocotyledonous plants, including members of Poaceae and naturally occurring bamboo such as *Dendrocalamus* spp.^7, 9, 39^. Corridors that reconnect forest patches but lack suitable forage may continue to channel elephants toward agricultural edges. In this context, corridor planning must avoid unintentionally increasing attraction to cultivated crops. Ecological trap theory provides a useful framework whereby animals preferentially select habitats that appear resource-rich but ultimately reduce fitness under altered environmental conditions^50, 51^. In agricultural mosaics, energy-rich crops may act as strong cues of food availability. If these cues increase habitat preference while simultaneously elevating the risk of conflict and associated injury or repeated translocation, the resulting fitness costs may outweigh short-term energetic gains. Under such conditions, landscapes may function as ecological trap, where elephants preferentially using high-risk agricultural areas despite the availability of safer natural forage in forests. Management should therefore differentiate forage strategies across the landscape by enhancing native forage within forest interiors while concentrating highly preferred forage at strategic locations to redirect feeding pressure away from agricultural areas, including palatable non-native species such as Napier grass. Aligning corridor design with elephants’ dietary ecology may reduce repeated crop use and promote long-term ecological stability and coexistence in multi-use tropical landscapes, particularly where movement routes intersect with concentrated food resources.

## Conclusion

This study provides insights into the dietary responses of Asian elephants across different landscapes in Peninsular Malaysia, utilising high-throughput DNA metabarcoding combined with comprehensive bioinformatics analysis. By integrating Relative Read Abundance (RRA) and Frequency of Occurrence (FOO) metrics, this study offers an accurate and detailed representation of elephant diets, providing deeper ecological insights into their foraging strategies across contrasting landscapes. Our findings reveal that elephants in development-logging landscapes (DLL) exhibit higher dietary diversity, driven by ongoing logging activities and resource scarcity. In contrast, elephants in oil palm-forest landscapes (OPFL) rely on a more specific diet, including many cultivated plant species. Statistical analyses illustrate significant dietary variations, demonstrating that landscape configuration is closely associated with patterns of resource use. The association between landscape configuration and different in dietary composition highlights the importance of coordinated land-use planning that integrates habitat preservation, connectivity and agricultural management to mitigate human–elephant conflict (HEC). Understanding the spatial and temporal variations in elephant diet is crucial for designing evidence-based conservation strategies that ensure the long-term sustainability of Asian elephant populations, particularly in human-modified landscapes. Lastly, this study reinforces the potential of DNA metabarcoding and bioinformatics to enhance the accuracy of ecological assessments, unveiling dietary patterns with greater precision. These findings contribute to wildlife conservation strategies while also emphasising FOO as a reliable metric in dietary studies.

## Data Availability

The data supporting the findings of this study have been deposited in the Knowledge Network for Biocomplexity (KNB) and are publicly available at the following DOI: (https://knb.ecoinformatics.org/view/doi:10.5063/F15X27DB) . All relevant data are included within the article. Additional materials are available from the corresponding author upon reasonable request.

## References

[1] Karuppannan, K. et al. Sex ratio and age structure patterns of Asian elephants from Peninsular Malaysia revealed by non-invasive surveys. *JAPS J. Anim. Plant. Sci*. **30**(6). (2020).

[2] Saaban, S. et al. Current status of Asian elephants in Peninsular Malaysia. *Gajah* **35** (1), 67–75 (2011).

[3] IUCN. *Elephas maximus*. *IUCN Red List. Threatened Species*. 2024, eT7140A45818198. 10.2305/IUCN.UK.2020-3.RLTS.T7140A45818198.en

[4] PERHILITAN. *Red List of Mammalsfor Peninsular Malaysia* (Department of Wildlife and National Parks (PERHILITAN) Peninsular Malaysia, 2017).

[5] Mohd-Radzi, N. H. S. et al. Determining the diet of wild Asian elephants (*Elephas maximus*) at human–elephant conflict areas in Peninsular Malaysia using DNA metabarcoding. *Biodivers. Data J.* **10**. (2022). 10.3897/BDJ.10.e89752 PMC9836633 36761586

[6] Saaban, S. et al. Viability and management of the Asian elephant (*Elephas maximus*) population in the Endau Rompin landscape, Peninsular Malaysia. *PeerJ.* **8**, e8209 (2020). 32002318 10.7717/peerj.8209 PMC6984340

[7] Abdullah-Fauzi, N. A. F. et al. Determining the dietary preferences of wild Asian elephants (Elephas maximus) in Taman Negara National Park, Malaysia based on sex and age using trnL DNA metabarcoding analysis. *Zool. Stud.* **61**. (2022). 10.6620/ZS.2022.61-60 PMC10061209 37007822

[8] Chen, J., Deng, X., Zhang, L., Bai, Z. & Wang, S. Diet composition and foraging ecology of Asian elephants in Xishuangbanna, China. *Acta Ecol. Sin.* **26**, 309–316 (2006).

[9] Koirala, R. K., Raubenheimer, D., Aryal, A., Pathak, M. L. & Ji, W. Feeding preferences of the Asian elephant (*Elephas maximus*) in Nepal. *BMC Ecol.* **16**, 1–9 (2016). 27855704 10.1186/s12898-016-0105-9 PMC5114758

[10] Birnie-Gauvin, K., Peiman, K. S., Raubenheimer, D. & Cooke, S. J. Nutritional physiology and ecology of wildlife in a changing world. *Conserv. Physiol.* **5** (1), cox030. 10.1093/conphys/cox030 (2017). 28740638 10.1093/conphys/cox030 PMC5516125

[11] Vancuylenberg, B. Feeding behaviour of the Asiatic elephant in south-east Sri Lanka in relation to conservation. *Biol. Conserv.* **12** (1), 33–54 (1977).

[12] Rode, K. D., Chiyo, P. I., Chapman, C. A. & McDowell, L. R. Nutritional ecology of elephants in Kibale National Park, Uganda, and its relationship with crop-raiding behaviour. *J. Trop. Ecol.* **22** (4), 441–449 (2006).

[13] Pyke, G. H. Optimal foraging theory: A critical review. *Annu. Rev. Ecol. Syst.* **15**, 523–575 (1984).

[14] Cao, Z. et al. Comparison and association of winter diets and gut microbiota using trnL and 16S rRNA gene sequencing for three herbivores in Taohongling, China. *Glob. Ecol. Conserv.* **53**, e03041 (2024).

[15] Ismail, N. A., Daud, U. N. S., Arazmi, N. F. N., Nor, S. M. & Mansor, M. S. Dietary shifts in Barn Swallow Hirundo rustica passage and wintering in Peninsular Malaysia: From the early to late migratory season. *Eur. J. Wildl. Res.* **71** (2), 1–12 (2025).

[16] Wadey, J. et al. Why did the elephant cross the road? The complex response of wild elephants to a major road in Peninsular Malaysia. *Biol. Conserv.* **218**, 91–98 (2018).

[17] Ong, L. et al. A., Asian elephants as ecological filters in Sundaic forests. *Front. Forests Glob. Change*. **6**, 1143633 (2023).

[18] Zafir, A. W. A. & Magintan, D. Historical review of human-elephant conflict in Peninsular Malaysia. *J. Wildl. Parks*. **31**, 1–19 (2016).

[19] Nor Hafisa, S. M. R. et al. Determining the diet of wild Asian elephants (*Elephas maximus*) at human–elephant conflict areas in Peninsular Malaysia using DNA metabarcoding. (2022). 10.3897/BDJ.10.e89752 PMC9836633 36761586

[20] Yamamoto-Ebina, S., Saaban, S., Campos-Arceiz, A. & Takatsuki, S. Food habits of Asian elephants Elephas maximus in a rainforest of northern Peninsular Malaysia. *Mamm. Study*. **41**(3), 155–161 (2016).

[21] Bokulich, N. A. et al. q2-longitudinal: longitudinal and paired-sample analyses of microbiome data. *MSystems.* **3** (6), 00219 – 00218 (2018). 10.1128/msystems 10.1128/mSystems.00219-18 PMC6247016 30505944

[22] Han, E. K., Cho, W. B., Tamaki, I., Choi, I. S. & Lee, J. H. Comparative mitogenomic analysis reveals gene and intron dynamics in Rubiaceae and intra-specific diversification in damnacanthus indicus. *Int. J. Mol. Sci.* **22** (13), 7237 (2021). 34281291 10.3390/ijms22137237 PMC8268409

[23] Martin Říhová, J., Gupta, S., Nováková, E. & Hypša, V. Fur microbiome as a putative source of symbiotic bacteria in sucking lice. *Sci. Rep.* **14** (1), 22326 (2024). 39333204 10.1038/s41598-024-73026-2 PMC11436785

[24] Arazmi, F. N., Ismail, N. A., Daud, U. N. S. & Mansor, M. S. DNA metabarcoding unveils habitat-linked dietary variation in aerial insectivorous birds. *Animals.* **15** (7), 974 (2025). 40218367 10.3390/ani15070974 PMC11987892

[25] Mansor, M. S., Halim, M. R. A., Abdullah, N. A. & Ramli, R. Barn Swallows Hirundo rustica in Peninsular Malaysia: urban winter roost counts after 50 years, and dietary segregation from house-farmed swiftlets Aerodramus sp. *Raffles Bull. Zool.* **68**. (2020).

[26] Finnegan, A., Sao, S. S. & Huchko, M. J. Using a chord diagram to visualize dynamics in contraceptive use: bringing data into practice. *Glob. Health Sci. Pract.* **7** (4), 598–605 (2019). 10.9745/GHSP-D-19-00205 PMC6927835 31818870

[27] Oksanen, J. vegan: Community Ecology Package. (2022). Available from: https://CRAN.R-project.org/package=vegan

[28] Jamaluddin, M. I. M. et al. Asian elephants involved in conflicts exhibit similar habitat use but travel farther than non-conflict individuals. *Glob. Ecol. Conserv.* **55**, e03228 (2024).

[29] Magioli, M. et al. P. M. d. B. Dietary expansion facilitates the persistence of a large frugivore in fragmented tropical forests. *Anim. Conserv.* **25**(4), 582–593 (2022).

[30] English, M., Ancrenaz, M., Gillespie, G., Goossens, B. & Nathan, S. Linklater. Foraging site recursion by forest elephants *Elephas maximus* borneensis. *Curr. Zool.* **60** (4), 551–559 (2014).

[31] Cabral de Mel, S. J. et al. Attitudes towards causes of and solutions to conflict between humans and Asian elephants. *Conserv. Sci. Pract.* **6** (11), e13238 (2024).

[32] Evans, L. J., Goossens, B., Davies, A. B., Reynolds, G. & Asner, G. P. Natural and anthropogenic drivers of Bornean elephant movement strategies. *Glob. Ecol. Conserv.* **22**, e00906 (2020).

[33] Ortega, J. & Eggert, L. *The Living Elephants: Evolutionary Ecology, Behavior, and Conservation, *vol. 85 (Oxford University Press, 2004).

[34] Alfred, R. et al. Home range and ranging behaviour of Bornean elephant (*Elephas maximus* borneensis) females. *PloS one*. **7** (2), e31400 (2012). 22347469 10.1371/journal.pone.0031400 PMC3275559

[35] Mohd Taher, T. et al. Characteristic of habitat suitability for the Asian elephant in the fragmented Ulu Jelai Forest Reserve, Peninsular Malaysia. *Trop. Ecol.* **62**, 347–358 (2021).

[36] Perhilitan. *National report on wildlife and human-elephant conflicts* (Department of Wildlife and National Parks Peninsular Malaysia, 2023).

[37] Chen, Y. et al. Predicting hotspots of human-elephant conflict to inform mitigation strategies in Xishuangbanna, Southwest China. *PLoS One*. **11** (9), e0162035 (2016). 27631976 10.1371/journal.pone.0162035 PMC5025021

[38] Jamaluddin, M. I. M. et al. Ecological corridors enhance adaptation success of translocated conflict elephants: A case study of a sub-adult male in Hulu Terengganu, Peninsular Malaysia. *Ecol. Solut. Evid. ***6**(3), e70049 (2025).

[39] Sukumar, R. *The Living Elephants: Evolutionary Ecology, Behavior, and Conservation* (Oxford University Press, 2003).

[40] Lim, T. & Campos-Arceiz, A. A review of human-elephant ecological relations in the Malay Peninsula: Adaptations for coexistence. *Diversity* **14** (1), 36 (2022).

[41] Schwarz, C., Johncola, A. & Hammer, M. Foraging ecology of semi-free-roaming Asian Elephants in Northern Thailand. *Gajah. ***52** (2020).

[42] Campos-Arceiz, A. & Blake, S. Megagardeners of the forest – the role of elephants in seed dispersal. *Acta Oecol.* **37**, 542–553 (2011).

[43] Berita Harian. 313 gajah liar dipindahkan sejak 2021, Kelantan tertinggi 104. Berita Harian. (2025). https://www.bharian.com.my/berita/nasional/2025/11/1472089/313-gajah-liar-dipindahkan-sejak-2021-kelantan-tertinggi-104

[44] BERNAMA. Perhilitan Laksana Operasi Bersepadu Translokasi Gajah Besar-besaran. BERNAMA News Agency. (2025). https://www.bernama.com/bm/news.php?id=2445027

[45] Harian Metro, 265 gajah liar dipindahkan di tujuh negeri, *Harian Metro*, (2025).

[46] Graham, M. D., Douglas-Hamilton, I., Adams, W. M. & Lee, P. C. The movement of African elephants in a human‐dominated land‐use mosaic. *Anim. Conserv.* **12** (5), 445–455 (2009).

[47] Ram, A.K., Yadav, H.K., & Rijal, A.P., Landscape predictors of human elephant conflicts in Chure Terai Madhesh Landscape of Nepal, *Environmental Challenges*, 7. 10.1016/j.envc.2022.100458 (2022).

[48] Beier, P. & Noss, R. F. Do habitat corridors provide connectivity? *Conserv. Biol.* **12**, 1241–1252 (1998).

[49] Haddad, N. M. et al. Habitat fragmentation and its lasting impact on Earth’s ecosystems. *Science* **348**, 6230 (2015). 10.1126/sciadv.1500052 PMC4643828 26601154

[50] Robertson, B. A. & Hutto, R. L. A framework for understanding ecological traps and an evaluation of existing evidence. *Ecology* **87**, 1075–1085 (2006). 16761584 10.1890/0012-9658(2006)87[1075:affuet]2.0.co;2

[51] Schlaepfer, M. A., Runge, M. C. & Sherman, P. W. Ecological and evolutionary traps. *Trends Ecol. Evol.* **17**, 474–480 (2002).

